# P-795. Comparison of Clinical Outcomes after Treatment of Urinary Tract Infections Caused by Serratia marcescens, Morganella morganii, and Providencia spp. with Antibiotics Susceptible to versus Stable against AmpC Hydrolysis

**DOI:** 10.1093/ofid/ofaf695.1005

**Published:** 2026-01-11

**Authors:** Yingsi Fang, Kendall Bell, Ryan W Chapin, Christopher McCoy, Matthew Gwiazdon

**Affiliations:** Beth Israel Deaconess Medical Center, Boston, Massachusetts; BIDMC, Boston, Massachusetts; Beth Israel Deaconess Medical Center, Boston, Massachusetts; Beth Israel Lahey Health System, Boston, Massachusetts; Beth Israel Lahey Health (Plymouth), Plymouth, MA

## Abstract

**Background:**

The 2024 IDSA Guidance on the Treatment of Antimicrobial-Resistant Gram-Negative Infections suggests selecting antibiotics according to susceptibility results to treat infections caused by *S. marcescens*, *M. morganii*, and *Providencia spp*. Although these organisms are thought to be at moderate to low risk of developing clinically significant AmpC expression, use of broad-spectrum beta-lactam antibiotics remains common even for urinary tract infections (UTIs). No study to date has compared the clinical outcomes for UTIs caused by these organisms in patients who initially received antibiotics susceptible to AmpC hydrolysis to those treated with AmpC-stable antibiotics.

**Table 1.**
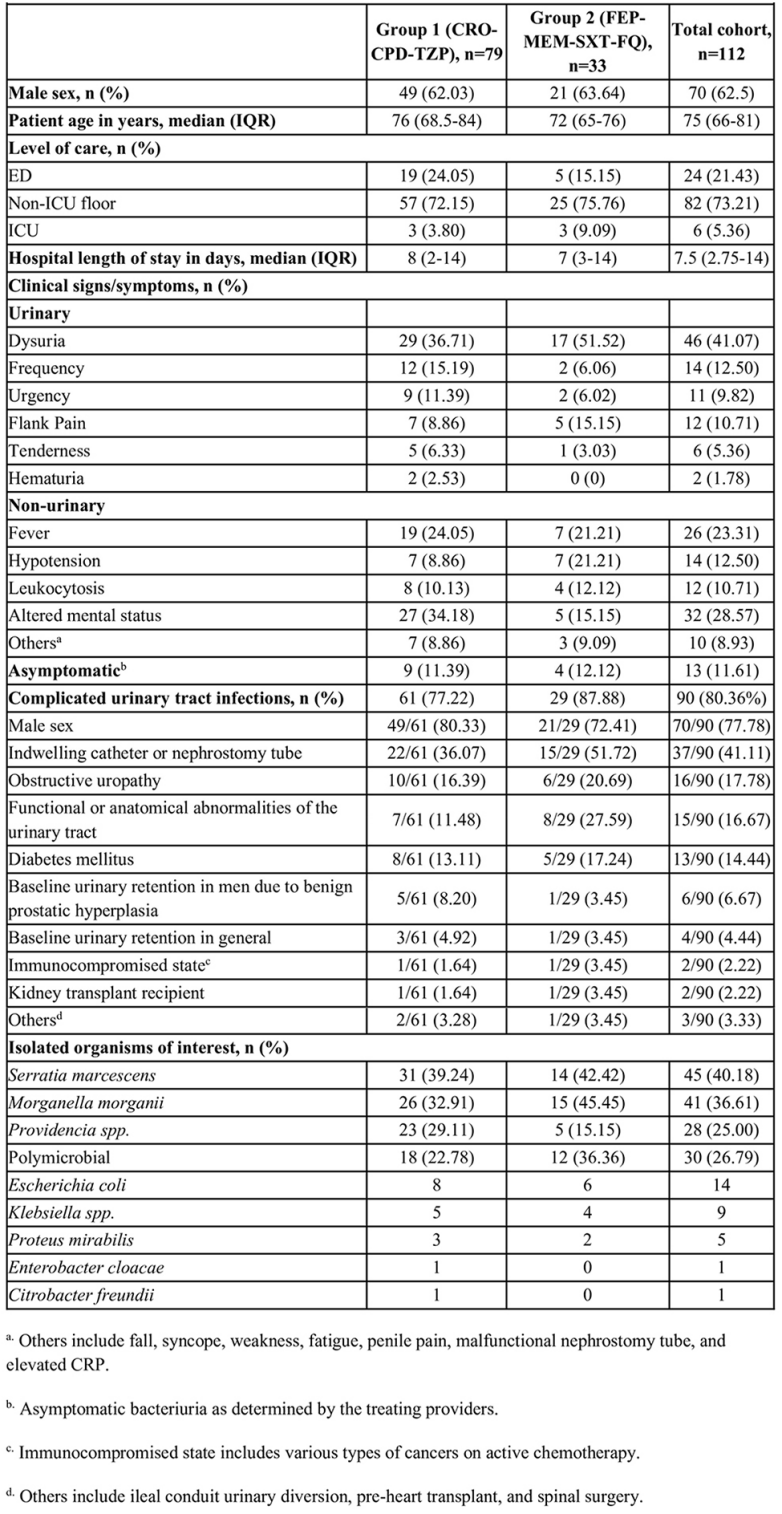
Baseline Characteristics

**Table 2.**
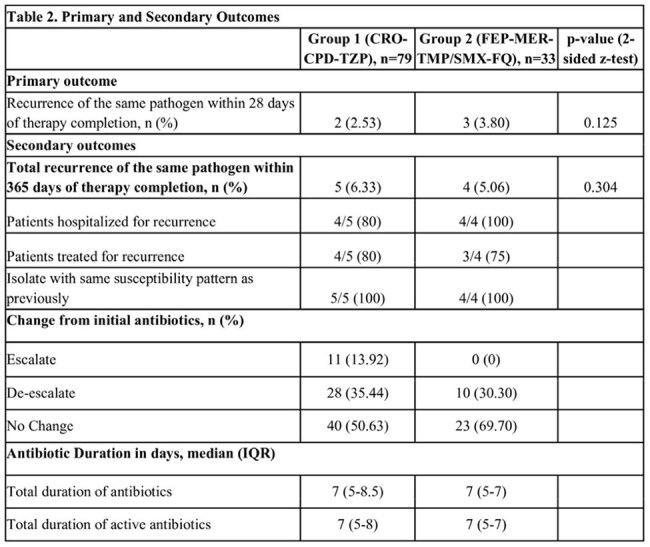
Primary and Secondary Outcomes

**Methods:**

This was a single-center retrospective study of non-pregnant patients > 18 years old with *S. marcescens*, *M. morganii*, or *Providencia spp.* isolated from a urine culture who received treatment with active antibiotics for at least 48 hours from October 2019 to June 2024. Patients with antibiotic use for other indications, polymicrobial urine cultures warranting broader antibiotic coverage, or isolates resistant to ceftriaxone were excluded. The primary outcome was recurrence of infection – defined as re-isolation of the same organism in a urine culture within 28 days of treatment.

**Results:**

112 unique patients met the inclusion criteria, Patients were divided into two cohorts—Group 1 consisted of 79 patients (70.5%) who received ceftriaxone (CRO), cefpodoxime (CPD), or piperacillin/tazobactam (TZP), and Group 2 had 33 patients (29.5%) receiving cefepime (FEP), meropenem (MEM), trimethoprim/sulfamethoxazole (SXT), or a fluoroquinolone (FQ). 90 patients (80.36%) met IDSA criteria for complicated UTI (Table 1). The primary outcome occurred in 2 patients (2.53%) in Group 1 and 3 patients (3.8%) in Group 2. The same organism was reisolated within a year of treatment in 5 patients from Group 1 and 4 patients from Group 2 – all of which had unchanged susceptibility patterns (Table 2).

**Conclusion:**

In this population of patients with UTI caused by organisms with low risk of AmpC expression, the observed rate of UTI recurrence was similar after initial treatment with antibiotics susceptible to AmpC hydrolysis compared to antibiotics stable against AmpC hydrolysis.

**Disclosures:**

All Authors: No reported disclosures

